# Comparative genomics and stress-responsive expression of the IMP gene family in *Sorghum bicolor*

**DOI:** 10.3389/fpls.2026.1941259

**Published:** 2026-09-10

**Authors:** Ling Zheng, Xiaoyu Zhou, Fei Wei, Yaqi Han, Jinbo Li, Jianming Han

**Affiliations:** Department of Biology, Luoyang Normal University, Luoyang, Henan, China

**Keywords:** abiotic stress response, comparative genomics, functional divergence, IMP gene family, *Sorghum*

## Abstract

Inositol monophosphatase (IMP) is a key enzyme in the inositol signaling pathway and ascorbic acid (AsA) biosynthesis, playing crucial roles in plant growth, development, and abiotic stress responses. Sorghum (*S. bicolor*) is an important cereal crop with remarkable drought and salinity tolerance, yet a systematic analysis of the IMP gene family in sorghum has been lacking. In this study, we identified eight IMP genes (*SbIMP-1–SbIMP-8*) in the sorghum genome. Phylogenetic analysis classified them into six subfamilies, consistent with rice and maize, suggesting a common ancestral origin. Gene structure and motif composition were highly conserved within each subfamily but varied among subfamilies, indicating functional divergence. Synteny analysis revealed strong collinearity between sorghum and maize with mostly one-to-one orthologous relationships, whereas complex one-to-many correspondences were observed with the polyploid *S. spontaneum*, highlighting lineage-specific expansion driven by whole-genome duplication. Promoter cis-element analysis identified numerous stress-related and hormone-responsive elements, including ABRE, DRE, MYB and MYC binding sites. Transcriptome and qRT-PCR analyses under salt (NaCl) and osmotic (PEG) stresses revealed distinct expression patterns: *SbIMP-1*, *SbIMP-3*, *SbIMP-6*, *SbIMP-7* and *SbIMP-8* acted as salt responders. In particular, *SbIMP-4* exhibited a strong salt-specific induction. Promoter enrichment of MYB and MYC binding sites was associated with rapid salt response, whereas the combination of DRE, ABRE and MYB sites in *SbIMP-4* likely underpinned its unique expression profile. This study provides preliminary insights into the expression profiles of SbIMP genes under salt and osmotic stresses, offering a theoretical foundation for molecular breeding aimed at improving abiotic stress response in sorghum and other crops.

## 1 Introduction

Plants have evolved elaborate molecular regulatory networks to adapt to environmental changes and ensure their survival. Nevertheless, various adverse environmental factors exert severe negative impacts on plant growth and development, often resulting in reduced crop yields and even plant death. These environmental stressors are generally categorized into biotic and abiotic stresses, with abiotic stresses encompassing a diverse array of adverse conditions such as drought, salinity, alkalinity, extreme temperatures and heavy metal pollution. Among these abiotic stressors, drought and high salinity are the most prominent and detrimental; they currently pose a grave threat to global food supply and the sustainability of agricultural systems (Bailey-Serres et al., 2019). In recent years, extreme climate events have further exacerbated the challenges faced by global agricultural production (Fedoroff et al., 2010). Recent statistics show that drought has caused billions of dollars in crop yield losses worldwide, and climate change is predicted to halve the availability of freshwater resources (Gupta et al., 2020). More importantly, an increasing area of arable land is suffering from salinization, while effective strategies to control the spread of soil salinization are still lacking (Jones et al., 2014). Against this backdrop, identifying and characterizing stress-tolerant genes in sorghum has become an urgent and crucial research priority for improving crop abiotic stress resistance, thereby safeguarding global food security and promoting the development of sustainable agriculture.

Sorghum acts as a staple food crop in many countries and is widely cultivated across tropical, subtropical and temperate regions. It serves as an essential source of food for human consumption, as well as a raw material for fiber production and biofuel generation, playing an indispensable role in human social and economic development (Xie and Xu, 2019; Ngara et al., 2021). For cereal and cash crops alike, the inositol signaling pathway is a key regulatory module in mediating growth, development and stress resistance, with phosphatases being the core functional components of this pathway. Phosphatases in the inositol signaling pathway are mainly classified into five categories: inositol polyphosphate 5-phosphatases (5PTases), suppressor of actin (SAC) phosphatases, SAL1 phosphatase/FIERY1 (FRY1) and its homologs, inositol monophosphatases (IMP), and phosphatase and tensin homologue deleted on chromosome 10 (PTEN)-related phosphatases (Jia et al., 2019). Studies have also found that inositol monophosphatase (IMPase) can catalyze the dephosphorylation of L-galactose 1-phosphate and participate in the biosynthesis of ascorbic acid (AsA), acting as a bifunctional enzyme with dual catalytic activities, which suggests that the products encoded by IMP genes may possess bifunctional characteristics in plants (Torabinejad et al., 2009).

In recent years, accumulating research evidence has further confirmed that IMP genes are involved in plant responses to abiotic stresses by regulating the synthesis of ascorbic acid. In tobacco, overexpression of the rice *OsIMP* gene can significantly enhance the activity of antioxidant enzymes in plants, thereby improving their tolerance to cold stress (Zhang et al., 2017). In *Cicer arietinum*, the AsA content in plants with overexpressed *CaIMP* gene is significantly higher than that in wild-type plants, indicating that this gene can enhance the comprehensive stress tolerance of plants through regulating the AsA metabolic pathway (Saxena et al., 2013). In *Gossypium hirsutum*, *GhIMP10D* specifically increases the content of ascorbic acid (AsA) and enhances the activity of antioxidant enzymes in plants, which can efficiently scavenge reactive oxygen species (ROS) accumulated in plants under alkaline stress, effectively maintain the intracellular redox balance, and thus significantly alleviate the oxidative damage and growth inhibition of cotton caused by alkaline stress (Fan et al., 2023).

Despite the critical roles of IMP genes in plant development and stress responses, no systematic analysis of this family has been conducted in sorghum, a crop renowned for its natural drought and salt tolerance. Sorghum’s intrinsic stress tolerance may involve regulatory mechanisms absent in stress-sensitive cereals (Mace et al., 2013), while its phylogenetic position bridging diploid and polyploid Poaceae lineages provides an ideal framework for dissecting whole-genome duplication-driven gene family evolution (Yang et al., 2024). This study aims to uncover stress-adaptive genetic features in sorghum that could inform translational breeding strategies across cereal crops.

In this study, we systematically identified and characterized the IMP gene family in sorghum and conducted a comprehensive comparative genomic analysis with representative Poaceae species, including maize, Miscanthus, and Saccharum. By integrating phylogenetic reconstruction, gene structure annotation, synteny network mapping, and cis-element profiling, we elucidated the evolutionary trajectory and potential regulatory mechanisms of this family. Furthermore, combining transcriptome data with qRT-PCR validation, we precisely dissected the spatiotemporal expression dynamics of *SbIMP* genes under salt and osmotic stresses. These findings not only provide a solid theoretical foundation for understanding the functional differentiation of IMP genes in sorghum but also identify key candidate genes for future molecular breeding aimed at enhancing crop resilience to abiotic stresses.

## 2 Materials and methods

### 2.1 Identification of the IMP proteins

In the present study, genomic and annotation data of various plant species were collected and subjected to subsequent analysis (**Supplementary Table 1**). The hidden Markov model profile of PF00459, retrieved from the InterPro database, was employed to conduct a search against the aforementioned protein sequences via the hmmsearch module integrated in HMMER software, for the purpose of identifying members of the inositol monophosphatase (IMP) family (Finn et al., 2011; Blum et al., 2025). The preliminary identified IMP gene family members were further validated through InterProScan software, and only those proteins harboring the conserved IMP domain were screened out for follow-up experiments (Jones et al., 2014). Additionally, the ProtParam tool on the Expasy online server was utilized to perform physicochemical property analysis of the confirmed IMP family members (Gasteiger, 2003).

### 2.2 Phylogenetic tree analysis

High-precision homology alignment of protein sequences was conducted using MAFFT (v7.505) (Katoh and Standley, 2013). The initial alignment results were further optimized with trimAl (v1.4.rev15) to remove poorly aligned regions and sequence gaps, which served to improve the signal-to-noise ratio for subsequent phylogenetic analyses (Capella-Gutiérrez et al., 2009). A phylogenetic tree was then constructed based on the refined alignment data using the FastTree program, and the final tree was visualized and annotated via the tvBOT platform (Price et al., 2009; Xie et al., 2023).

### 2.3 Gene structure analysis

Genetic annotation information of the sorghum IMP gene family members, including the distribution and structural characteristics of introns and exons, was extracted from the corresponding gff file. All the aforementioned structural information was then visualized by means of the TBtools software (Chen et al., 2020).

### 2.4 Chromosomal localization, collinearity and duplication event analysis

Chromosomal localization data of IMP gene family members, including specific chromosomal locations and genomic start/end coordinate information, was extracted from genome annotation files in GFF3 format. Collinearity analysis of the IMP gene family was subsequently performed using the MCScanX software, and both chromosomal localization patterns and collinear relationships of the genes were visualized via Circos software (Wang et al., 2012; Krzywinski et al., 2009).

In addition, the duplicate_gene_classifier module embedded in MCScanX was adopted to identify gene duplication events within the IMP family (Wang et al., 2012). The non-synonymous substitution rate (Ka) and synonymous substitution rate (Ks) of the identified duplicated gene pairs were calculated through a combined application of the ParaAT workflow and KaKs_Calculator software (Zhang et al., 2012; Wang et al., 2010).

### 2.5 Analysis of promoter *cis*-acting elements

The 2000 bp upstream regulatory sequences of the coding regions of sorghum IMP family genes were extracted from the genomic sequence file, and these sequences were submitted to the PLANTCARE online server for cis-acting element prediction and analysis (Lescot, 2002).

### 2.6 Transcriptome data analysis

The RNA-seq reads used in this section were obtained from a publicly available dataset deposited in the NCBI Sequence Read Archive (SRA; BioProject accession PRJNA1159475; Yim et al., 2026) and were re-analyzed in the present study. In the original study, Yim et al. (2026) sequenced transcriptomes of Sorghum bicolor cv. ‘Hwanggeumchal’ seedlings subjected to multiple abiotic stresses and sampled at 6, 12, and 24 h after treatment. The transcriptome sequencing was performed in that study; the authors of the present study did not perform the sequencing experiment themselves. First, SRA-format files were converted into FASTQ format using the fastq-dump utility included in the SRA Toolkit. RNA-seq reads were aligned to the S. bicolor reference genome (BTx623, v3.1) with Hisat2 (Kim et al., 2015). The resulting SAM files were converted to BAM format using samtools for subsequent analysis (Li et al., 2009). Transcript assembly and isoform expression quantification were performed based on the aligned reads using StringTie software (Pertea et al., 2015). Gene-level raw read counts were calculated by featureCounts, which assigns sequencing reads to corresponding genomic features according to the reference annotation file and generates a raw count matrix (Liao et al., 2014). Ultimately, the raw count matrix was normalized to FPKM (Fragments Per Kilobase of transcript per Million mapped reads) values using the R programming language to standardize gene expression levels for comparative analysis.

### 2.7 RNA isolation and quantitative reverse transcription PCR assay

Seeds of *S. bicolor* (cv. Kansas Collier) were germinated in an artificial climate incubator under a photoperiod of 16 h light/8 h dark, with the temperature regulated at 27°C for the light period and 22°C for the dark period. Three-week-old seedlings were subjected to salt stress treatment with 200 mM NaCl and 20% PEG 6000, and leaf samples were collected at 0 h, 6 h, 12 h and 24 h post-stress initiation. All collected samples were immediately snap-frozen in liquid nitrogen and stored at -80°C for subsequent RNA extraction.

Total RNA was extracted from the frozen leaf samples using the Vazyme RNA Isolation Kit (Cat. No. RC411-C1). First-strand complementary DNA (cDNA) was synthesized from the purified total RNA with the Vazyme Reverse Transcription Kit (Cat. No. R223-01). Quantitative real-time PCR (qRT-PCR) was performed with ChamQ Universal SYBR qPCR Master Mix (Vazyme, Cat. No. Q711--02). SbUBQ10 was used as the reference gene for data normalization, and the relative expression levels of target genes were calculated by the 2^-ΔΔCT^ method (Livak and Schmittgen, 2001). The specific primer sequences used for qRT-PCR are listed in **Supplementary Table 2**.

The 200 mM NaCl and 20% (w/v) PEG6000 treatments were selected to elicit robust and reproducible transcriptomic responses; these concentrations are consistent with those commonly used in previous sorghum abiotic-stress transcriptomic studies. The present work focuses on expression-level responses; stress-tolerance phenotypes (e.g., survival, growth) were not assessed and remain outside the scope of this study.

### 2.8 Statistical analysis

For quantitative reverse transcription PCR (qRT-PCR) assays, all experimental data are presented as the mean ± standard deviation (SD). Each assay was performed with at least three independent biological replicates, and statistical analyses of the data were conducted using one-way analysis of variance (one-way ANOVA).

## 3 Results

### 3.1 Quantitative distribution and duplication patterns of the plant IMP gene family

**Figure 1:**
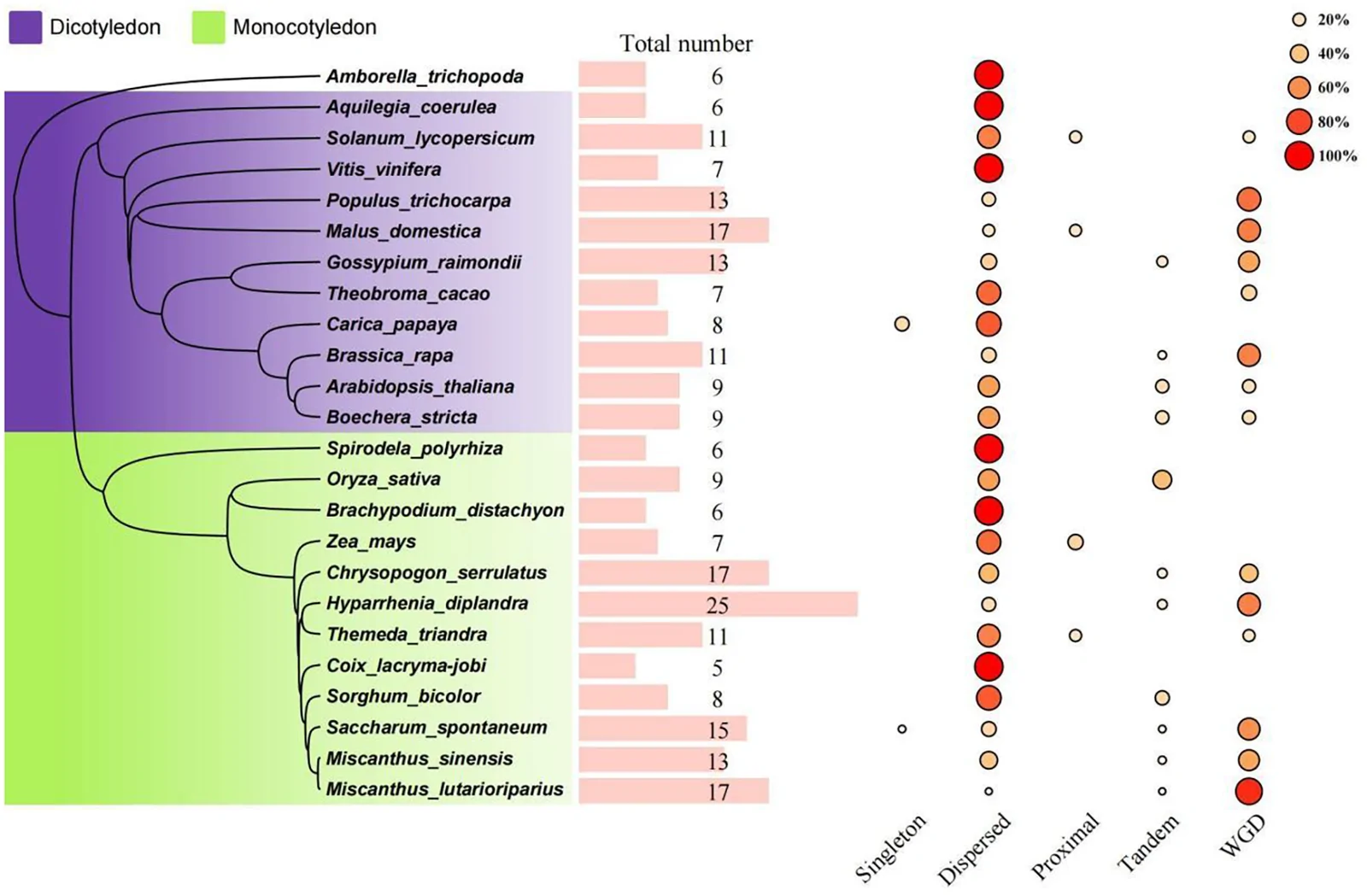
Gene number and duplication patterns of the IMP gene family across representative plant species. The phylogenetic tree of the species was obtained from timetree website, the purple and green box represent dicotyledons and monocotyledons, respectively. The middle bar chart shows the total number of IMP genes identified in each species. The right dot plot illustrates the proportional contribution of five gene duplication modes, with dot color intensity representing the relative percentage (ranging from 20% to 100%).

IMP genes are widely distributed across a diverse range of plant species (**Figure 1**). The basal angiosperm Amborella trichopoda harbors six IMP genes. Among dicotyledonous plants, the number of IMP genes ranges from 6 to 17, with Malus domestica possessing the largest repertoire (17 genes) and *Aquilegia coerulea* the smallest (6 genes). In monocotyledonous plants, the gene count varies from 5 to 25; *Hyparrhenia diplandra* contains the maximum number of IMP genes (25), while *Coix lacryma-jobi* has the minimum (5) (**Supplementary Table 1**). Interestingly, *S. bicolor* encodes 8 IMP genes, placing it at a moderate level among monocots.

With respect to the five gene duplication modes, dispersed duplication exhibits the highest proportion across most species, representing the primary mechanism driving the expansion of the IMP gene family, followed by whole-genome duplication (WGD). This evolutionary pattern is particularly prominent in gramineous crops such as sorghum, maize, and rice. In contrast, singleton, proximal, and tandem duplications account for relatively low proportions in the majority of species and only represent the dominant expansion mode in a few taxa. The significant variations in IMP gene number and duplication patterns across different species reflect the unique adaptive strategies that have evolved within their respective ecological niches.

### 3.2 Phylogenetic relationship analysis of IMP proteins

**Figure 2:**
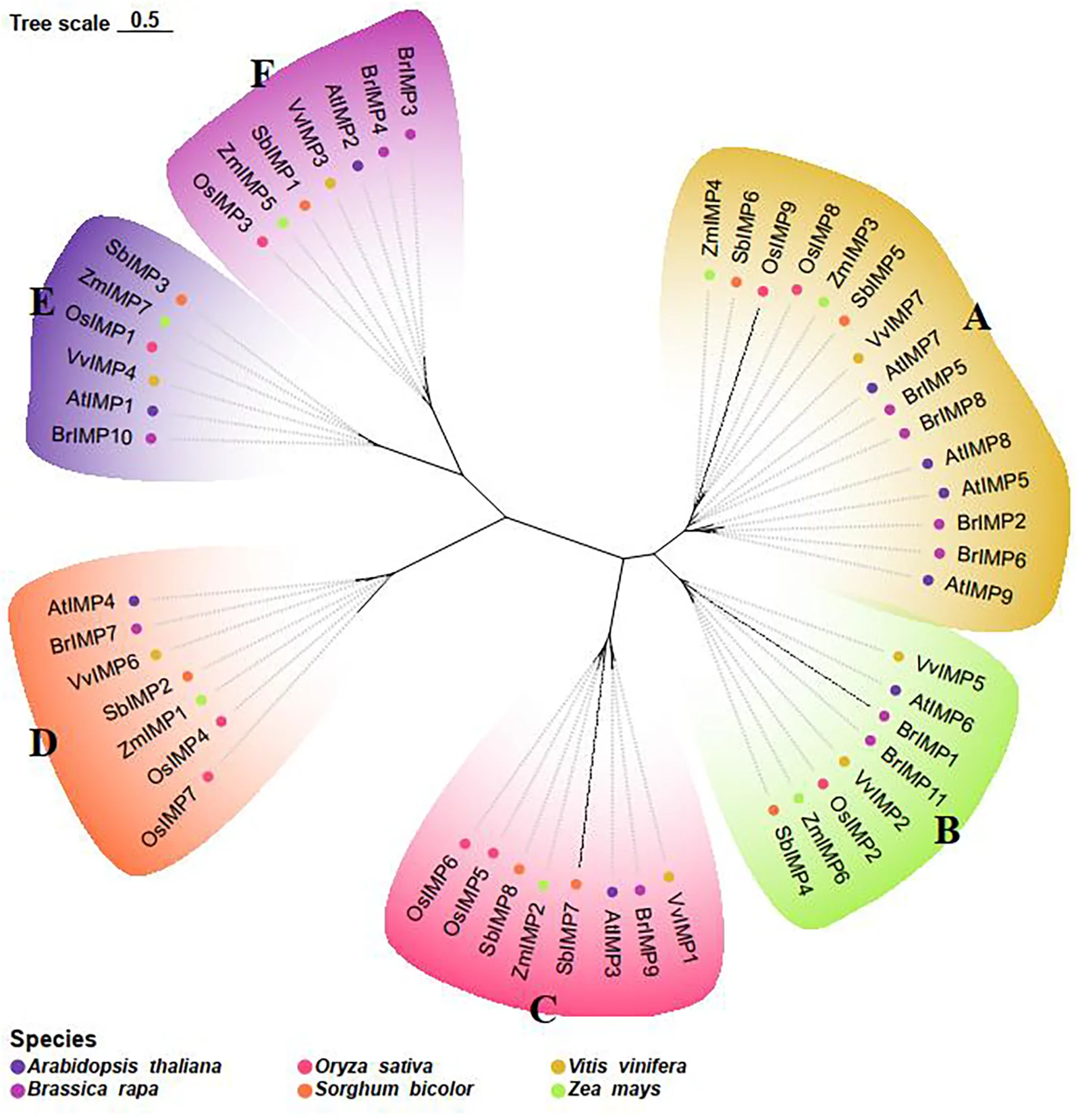
Phylogenetic analysis of IMP proteins. The tree was divided into six subfamilies **(A-F)**, indicated by distinct colored backgrounds. IMP proteins from different species are indicated by dots of various colors.

The IMP family was surveyed across 24 plant species at the genome-wide level (**Figure 1**), which established the family-wide distribution and evolutionary context. To resolve the orthology of each SbIMP subfamily in detail, a focused phylogenetic tree was then constructed with *S. bicolor* and five representative species (**Figure 2**). These six species were selected to include the grass representatives most closely related to sorghum (*O. sativa* and *Z. mays*) for reliable orthology inference, together with representative eudicots (A. thaliana, B. rapa, and *V. vinifera*) as taxonomic contrasts; including all 24 species surveyed in **Figure 1** would introduce long-branch noise from distantly related taxa and reduce topological resolution. The phylogenetic tree was divided into six subfamilies (designated A–F), with no species-specific independent clades formed (**Figure 2**). All SbIMP members formed tight sister clades with their orthologs from the closely related gramineous species *Z.mays* (*ZmIMP*) and *O. sativa* (*OsIMP*), indicating strong homology within the grass family. For instance, *SbIMP-2* clustered with *ZmIMP-1* and *OsIMP-4* in subfamily D, *SbIMP-4* clustered with *ZmIMP-6* and *OsIMP-2* in subfamily B, and *SbIMP-5* clustered with *ZmIMP-3* and *OsIMP-8* in subfamily A. Notably, *SbIMP-8*, *SbIMP-6* and other members were closely adjacent to IMP genes from maize and rice in subfamily C and A, reflecting high functional conservation of IMP genes between sorghum and other gramineous crops.

### 3.3 Conserved motif composition and gene structure analysis of *SbIMP* members

**Figure 3:**
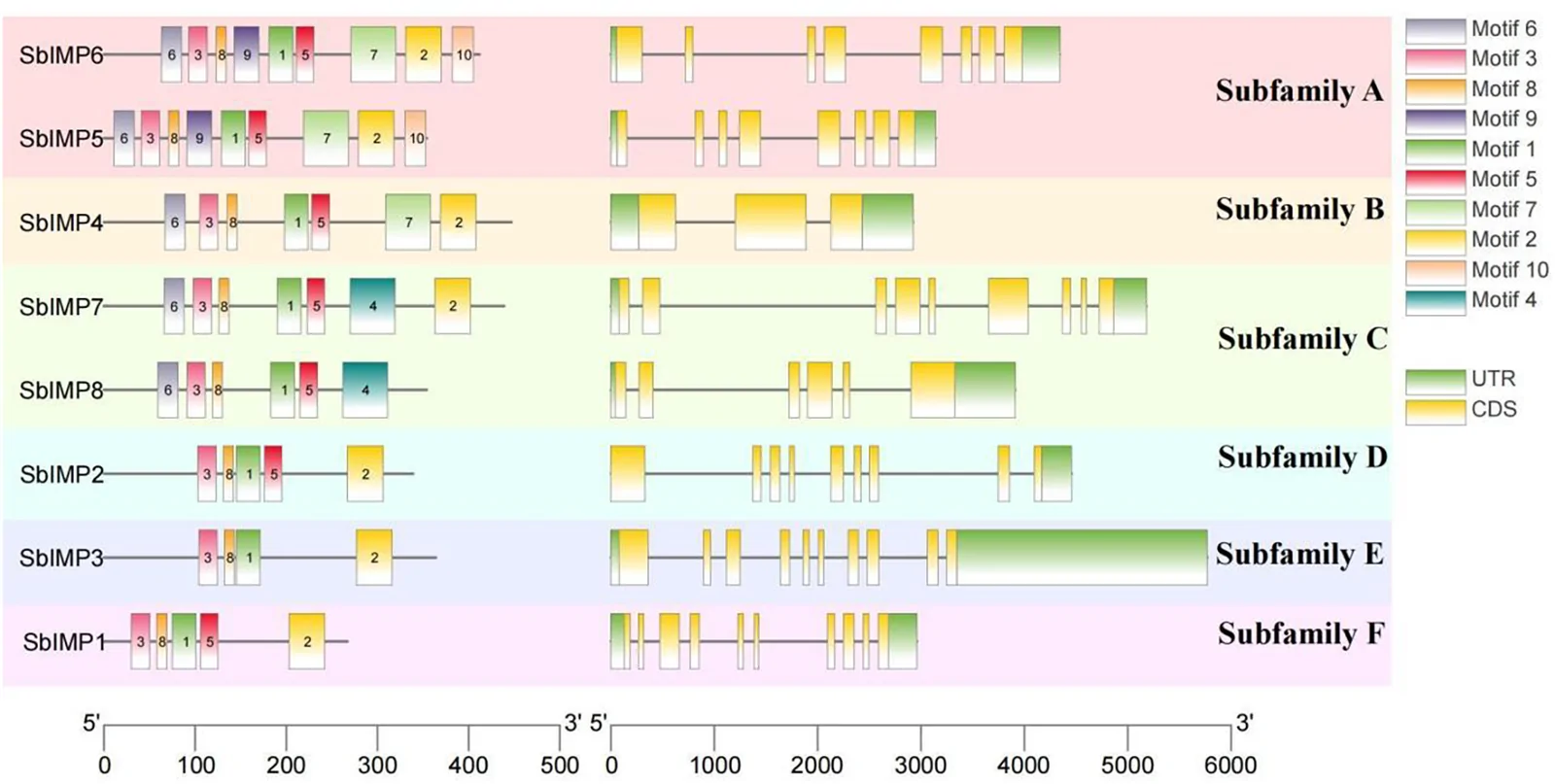
Conserved motif composition and gene structural analysis of SbIMP members. The left panel shows the distribution of 10 conserved motifs, with each colored box representing a specific motif. The right panel displays the gene structures, where yellow boxes indicate coding sequences (CDS), green boxes represent untranslated regions (UTR), and black lines denote introns. The scale bars at the bottom indicate the length of the protein (left) and gene (right) in base pairs.

Analysis of conserved motifs and gene structures revealed a high degree of uniformity within each SbIMP subfamily (**Figure 3**). Specifically, members clustered in the same subfamily shared nearly identical motif compositions, arrangement orders, and exon-intron architectures. For instance, *SbIMP-5* and *SbIMP-6* in Subfamily A exhibited an identical motif organization, while *SbIMP-7* and *SbIMP-8* in Subfamily C both contained the specific insertion of Motif 4. This structural conservation strongly corroborates the robustness of our phylogenetic classification. In contrast, distinct structural divergences were observed among different subfamilies, including the loss of specific N-terminal motifs (e.g., Motif 6 and 9 in Subfamilies D-F), the acquisition of unique motifs (e.g., Motif 4 in Subfamily C), and dramatic variations in the length of untranslated regions (e.g., the exceptionally long 3’ UTR in Subfamily E). These structural disparities suggest that functional specialization has occurred among SbIMP subfamilies during evolution.

### 3.4 Interspecific synteny analysis between sorghum and other Poaceae crops

**Figure 4:**
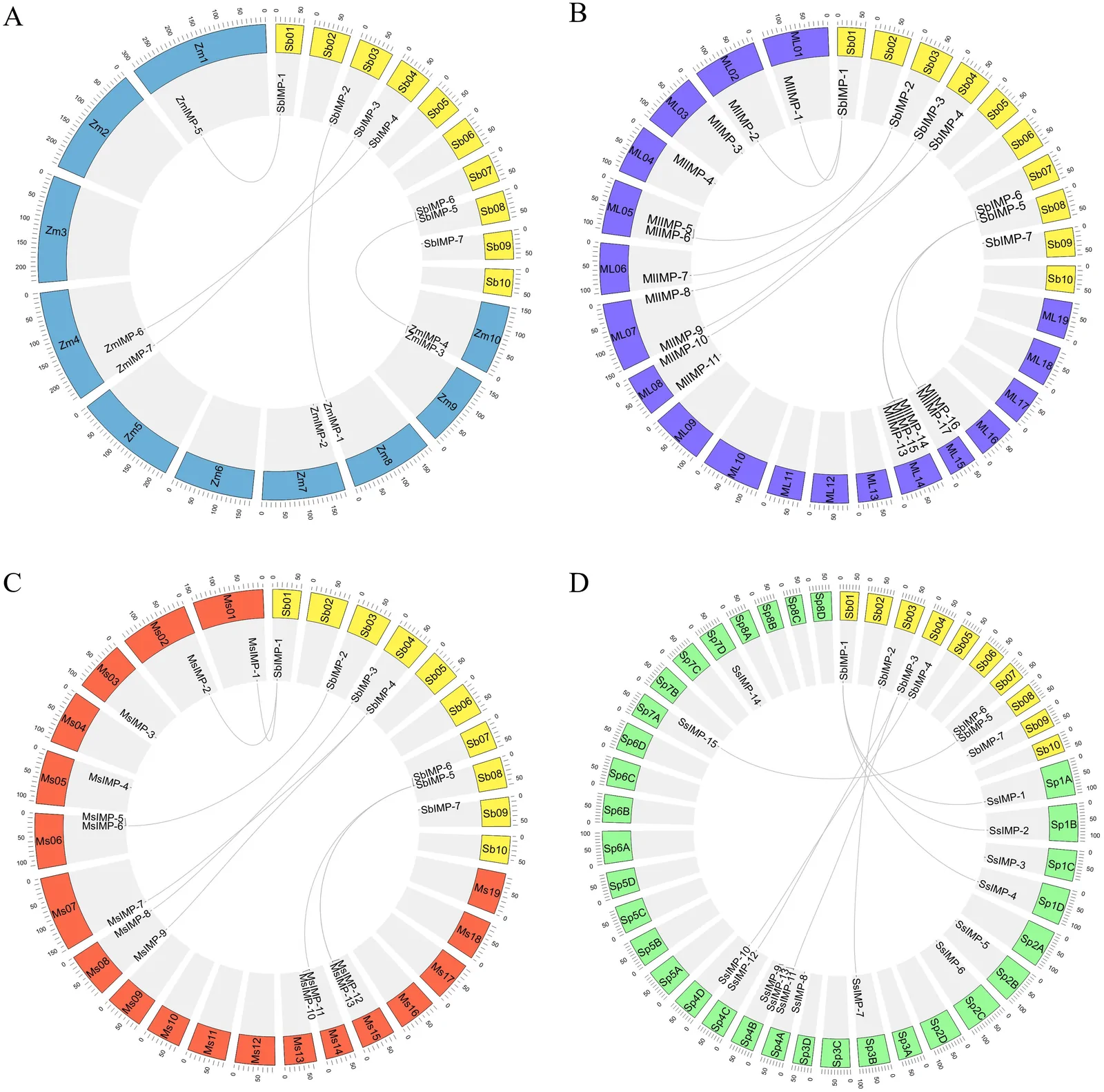
Synteny analysis of the IMP gene family between sorghum (*S. bicolor*) and four Poaceae species. **(A)** *S. bicolor* vs. maize (*Z. mays*). **(B)** *S. bicolor* vs. *M. lutarioriparius*. **(C)** *S. bicolor* vs. *M. sinensis*. **(D)** *S. bicolor* vs. *S. spontaneum*. Yellow blocks represent *S. bicolor* chromosomes (labeled Sb01–Sb10) in all panels. Blue blocks represent *Z. mays* chromosomes (Zm1–Zm10) in **(A)**. Purple blocks represent *M. lutarioriparius* chromosomes in **(B)**. Orange blocks represent *M. sinensis* chromosomes in panel **(C)**. Green blocks represent *S. spontaneum* chromosomes in **(D)**. Gray arcs connect syntenic IMP gene pairs between genomes.

To investigate the evolutionary history of the IMP gene family in sorghum, we performed inter-species collinearity analyses with maize, *Miscanthus lutarioriparius*, *M. sinensis*, and *S. spontaneum* (**Figure 4**). The results revealed a highly conserved collinear relationship between sorghum and the diploid relative maize, characterized by predominantly one-to-one or simple correspondence patterns (e.g., *SbIMP-1* vs. *ZmIMP-5*). However, *ZmIMP-2* in maize lacked an obvious collinear counterpart in the sorghum genome, suggesting it may represent a maize-specific expansion or a gene lost in sorghum. In contrast, while sorghum shared a basic collinear framework with *M. lutarioriparius* and *M. sinensis*, these species exhibited widespread “one-to-many” correspondences (e.g., *SbIMP-7* mapping to multiple *MlIMP/MsIMP* genes) and possessed numerous IMP genes (such as *MlIMP-2*~*4*, *7*~*8*, *12*~*13* and *MsIMP-2*~*4*, *10*) without collinear matches in sorghum, indicating independent gene duplication events and lineage-specific expansions following their divergence. Most significantly, the collinearity network between sorghum and the polyploid *S. spontaneum* was the most complex: single or few sorghum IMP genes often corresponded to multiple homologous copies distributed across different chromosomes in *S. spontaneum* (e.g., *SbIMP-2/3/4* mapping to several *SsIMP* genes), while *S. spontaneum* also harbored many IMP genes (e.g., *SsIMP-1*~*6*, *14*~*15*) lacking collinear links to sorghum, vividly illustrating the dramatic expansion of this gene family driven by whole-genome duplication and highlighting its potential for functional diversification. Collectively, these findings delineate a complex evolutionary trajectory for the IMP gene family across Poaceae, ranging from conservation to dynamic expansion, accompanied by specific gene loss or neo-functionalization events.

### 3.5 Cis-acting element analysis of *SbIMP* gene promoters

**Figure 5:**
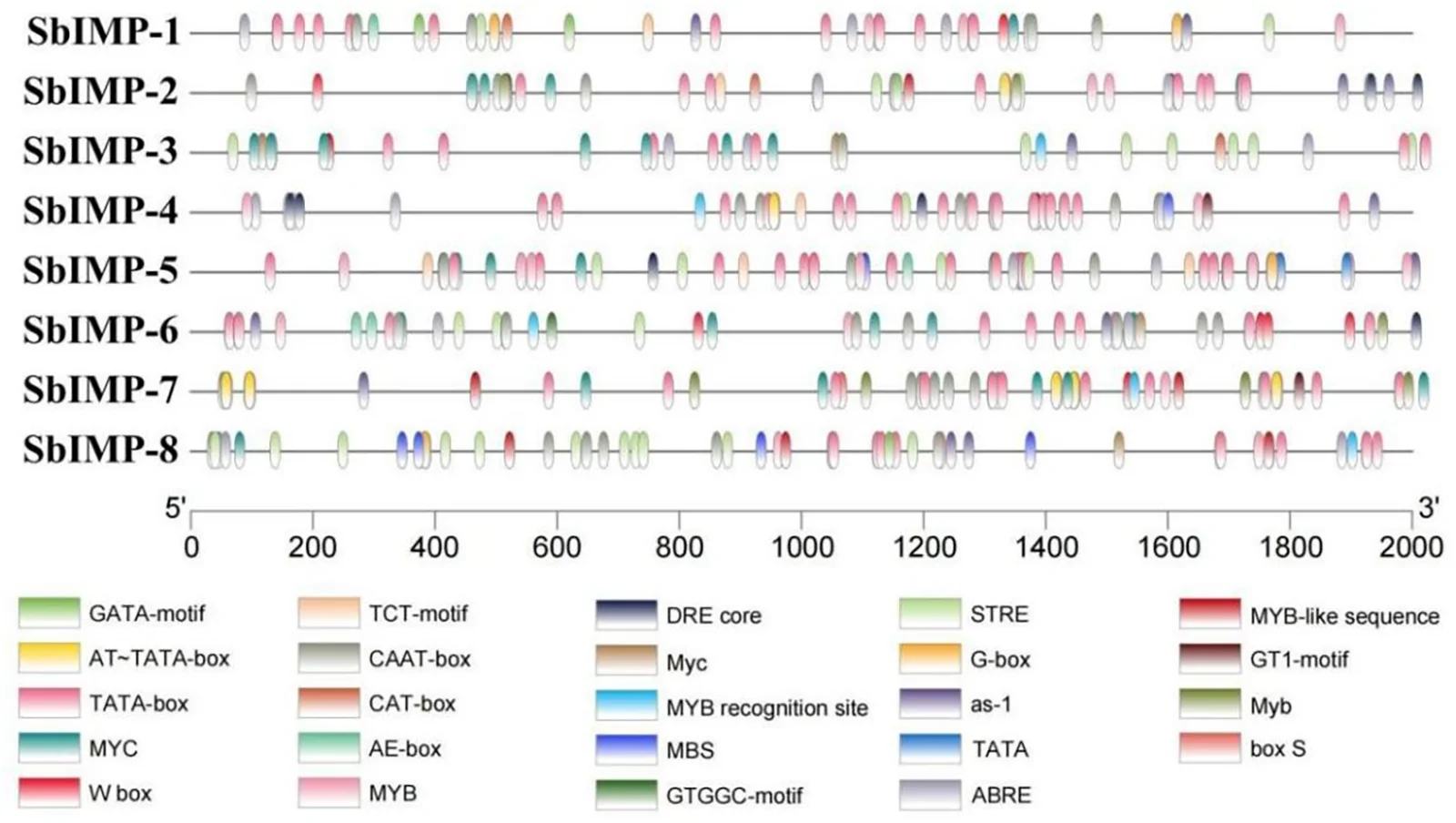
Distribution of cis-acting elements in the promoter regions of sorghum IMP genes. The schematic diagram illustrates the types and positions of predicted cis-acting regulatory elements within the 2000 bp upstream sequence. Each colored box represents a specific cis-element, as defined in the legend below. The horizontal axis indicates the nucleotide position from the 5’ end (0 bp) to the 3’ end (2000 bp).

To elucidate the transcriptional regulatory mechanisms of the sorghum IMP gene family, we systematically analyzed cis-acting elements within the 2000 bp upstream promoter regions (**Figure 5**). The results revealed that all eight *SbIMP* genes harbor a diverse array of cis-elements, indicating control by a complex regulatory network: in addition to universally present core promoter elements (such as TATA-box and CAAT-box), the promoters are widely populated with hormone-responsive elements (including ABA-responsive ABRE, jasmonate/defense-related MYC and MYB binding sites, and gibberellin-associated GATA-motif), suggesting deep involvement in hormone-mediated stress responses and developmental processes; furthermore, the identification of numerous environmental signal-responsive elements (such as drought-inducible DRE core and various light-responsive elements like G-box, GT1-motif, and AE-box) highlights the high sensitivity of *SbIMP* gene expression to external abiotic stresses (e.g., drought and light fluctuations); notably, significant variations in the types and numbers of elements exist among different genes (with *SbIMP-7* and *SbIMP-8* being particularly rich), implying specificity in their spatiotemporal expression patterns and functional differentiation, whereby individual members may respond to distinct internal signals or environmental stimuli to execute unique biological roles.

To correlate promoter architecture with expression dynamics, we quantified the four main stress-responsive cis-elements (ABRE, DRE, MYB, and MYC) in each SbIMP promoter (**Supplementary Table 4**). Early salt-responsive genes (*SbIMP-1/3/6/7/8*) showed variable MYC enrichment (1–9 copies), with SbIMP-3 and SbIMP-6 containing the highest MYC counts, whereas late osmotic responders (*SbIMP-2/5*) exhibited higher MYB abundance (8–10 copies). Notably, the salt-specific strong responder *SbIMP-4* uniquely harbored a combination of ABRE (4), DRE (3), and MYB (7) sites, a configuration absent from other members. These associations suggest that the combinatorial composition, rather than total element count, correlates with the divergent temporal response patterns among SbIMP genes.

### 3.6 Transcriptome-based expression profiling of *SbIMP* genes under abiotic stress

**Figure 6:**
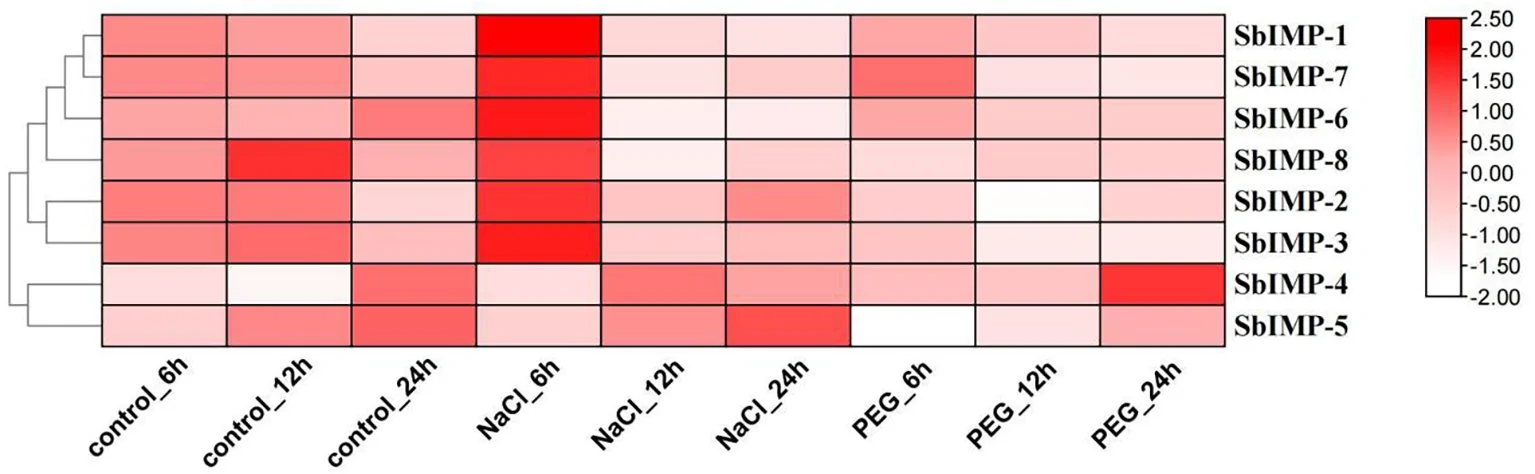
Heatmap of row-normalized expression profiles for *SbIMP* genes under salt and osmotic stress. The color scale represents the log2-transformed fold change relative to the mean expression of each gene, with red indicating upregulation and white indicating downregulation. Data are from the publicly available RNA-seq dataset (NCBI BioProject PRJNA1159475; Yim et al., 2026) and were re-analyzed in the present study.

In the re-analyzed public transcriptome, the *SbIMP* genes showed only modest changes in expression under NaCl or PEG treatment; no gene deviated from its mean expression by more than approximately 2-fold in any condition (**Figure 6**). *SbIMP*-*1*, *SbIMP*-*3*, *SbIMP-7*, and to a lesser extent *SbIMP-2* reached their highest relative expression at 6 h after NaCl treatment before declining, with *SbIMP-7* additionally showing pronounced downregulation at 12–24 h under both stresses. *SbIMP-4* displayed a relatively late response, with its highest relative expression at 12 h NaCl and 24 h PEG. *SbIMP-5*, *SbIMP-6*, and *SbIMP-8* remained largely stable across all conditions.

### 3.7 qRT-PCR analysis of *SbIMP* genes under abiotic stress

**Figure 7:**
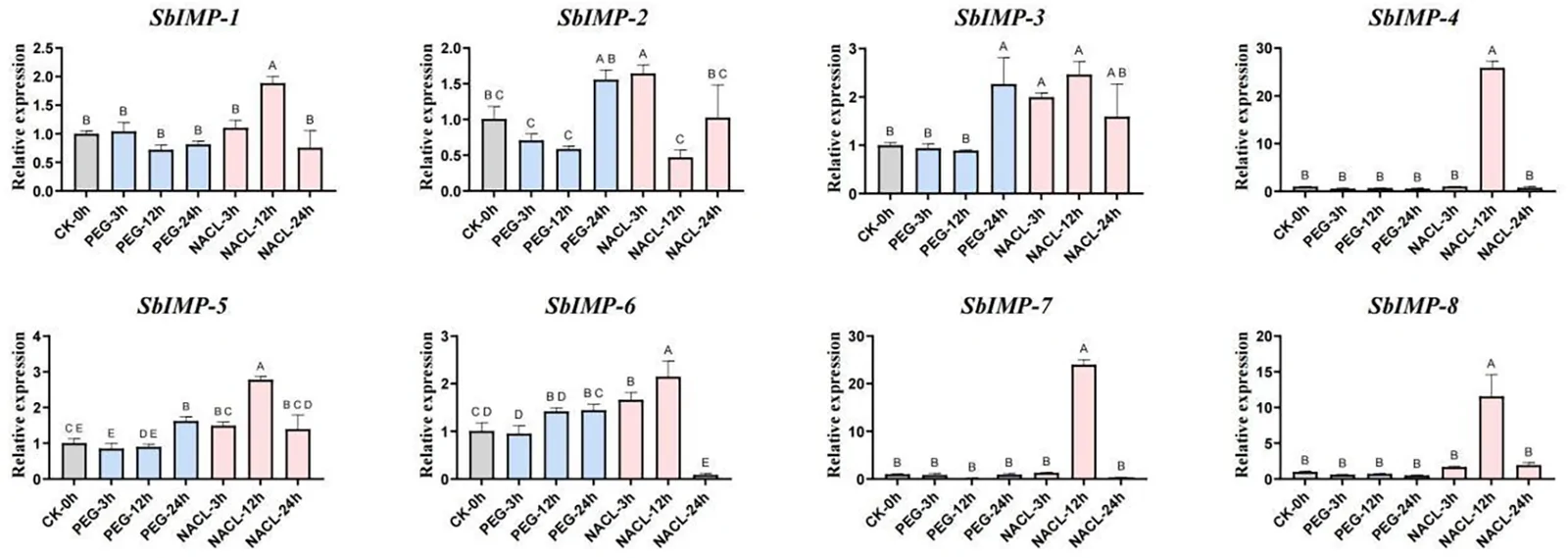
Expression analysis of *SbIMP* genes under salt and osmotic stress by qRT-PCR. Error bars represent standard deviation from three biological replicates. Different letters above bars indicate statistically significant differences (p < 0.05) among treatments for each gene.

To verify the reliability of transcriptome data and precisely resolve the expression dynamics of *SbIMP* genes under abiotic stress, we employed quantitative real-time PCR (qRT-PCR) to measure the relative expression levels of eight *SbIMP* genes at various time points following NaCl and PEG treatments (**Figure 7**). The results showed that the expression patterns of all tested genes were highly consistent with the transcriptome analysis, where in *SbIMP-1*, *SbIMP-3*, *SbIMP-6*, *SbIMP-7*, and *SbIMP-8* exhibited strong induction by salt stress, with most peaking at 12 hours, while *SbIMP-4* showed the strongest upregulation among all family members at 12 hours of salt stress, demonstrating strong salt specificity; in contrast, *SbIMP-2* and *SbIMP-5* primarily responded to osmotic stress, showing significant upregulation after 24 hours of PEG treatment; certain genes such as *SbIMP-2* responded to early signals of multiple stresses, and *SbIMP-6* displayed a continuous upward trend under both stresses; these findings not only confirmed the accuracy of the transcriptome data but also revealed fine-tuned temporal dynamics and functional differentiation among SbIMP family members in response to abiotic stress, suggesting that specific members acting as potent responders may participate in sorghum stress responses; direct functional validation is required to establish their contributions to stress response. The peak times and response magnitudes differed between the two datasets, which is expected because **Figures 6**, **7** are based on independent experiments using different genotypes (cv. Kansas Collier vs. cv. Hwanggeumchal) and different quantification metrics. Nevertheless, both approaches consistently identified *SbIMP-4* as the most strongly salt-induced member, supporting the robustness of our conclusions.

## 4 Discussion

As global warming accelerates the frequency of extreme heatwaves, droughts, and soil salinization, the urgent need to enhance crop resilience has become a paramount research priority. Sorghum is already recognized as a vital global resource, underpinning food security for over 500 million people in arid and semi-arid regions (Xin et al., 2021; Mace et al., 2013) and serving as a cornerstone of the renewable energy sector due to its unique versatility in producing grain, forage, sugar, and fiber (Gill et al., 2014). However, to sustain and expand this critical role amidst increasingly hostile climatic conditions, further improving the inherent abiotic stress resilience of sorghum is essential. Unlocking the genetic mechanisms behind its resilience is therefore key to ensuring its continued productivity and utility in a changing world.

Therefore, further mining of key stress-resistance genes in sorghum is essential for safeguarding yield stability under adverse conditions. Previous studies have established that inositol monophosphatase (IMPase) serves as a critical enzyme in the ascorbic acid (AsA) biosynthetic pathway and may enhance plant stress tolerance by modulating the AsA metabolic network. Functional characterizations of IMP genes in diverse species, including tobacco, rice, chickpea, and upland cotton, have consistently demonstrated their pivotal regulatory roles in response to abiotic stresses (Zhang et al., 2017; Saxena et al., 2013; Fan et al., 2023). However, despite these widespread functional insights in other crops, systematic analysis of the IMP gene family in sorghum remains notably scarce, limiting our comprehensive understanding of its potential value in sorghum stress resilience mechanisms.

In this study, we systematically identified eight *SbIMP* genes in the sorghum genome, a number that reflects a relatively compact family size compared to polyploid relatives but suggests lineage-specific retention within the Poaceae. Phylogenetic analysis classified these eight members into six distinct subfamilies (Subfamily A–F), a topology highly congruent with those observed in rice and maize, suggesting a common ancestral origin prior to the monocot-dicot divergence. Recently, comparative genomic studies in Poaceae have provided important evolutionary context for cross-species gene family analyses (Grass Phylogeny Working Group III, 2025).

Synteny analysis revealed contrasting evolutionary trajectories among Poaceae IMP genes: sorghum maintained predominantly one-to-one collinearity with diploid maize, whereas extensive one-to-many correspondences were observed with polyploid *S. spontaneum* (**Figure 4**). This pattern highlights the profound impact of recent polyploidization events in the Saccharum lineage on gene family diversification. Notably, similar WGD-driven expansion has been reported for other gene families in Poaceae; for example, the expansion of the TLP gene family in representative Poaceae lineages was also significantly influenced by WGD (Chen et al., 2023), suggesting that this mechanism is common in the evolution of grass gene families. Furthermore, the high conservation of exon-intron structures and motif compositions within each of the six subfamilies reinforces the reliability of our phylogenetic classification, implying that members of the same clade have retained similar biochemical functions, while structural variations between subfamilies suggest potential functional divergence during evolution. Although rice was not included in the inter-species synteny analysis, the strong sorghum–maize collinearity reported here (**Figure 4**) reflects the conserved ancestral grass karyotype that rice also shares (Paterson et al., 2009). Consistent with this, every SbIMP member clustered with its rice (OsIMP) and maize (ZmIMP) orthologs in the phylogenetic analysis (**Figure 2**). Together, these observations indicate that the genomic organization and sequence conservation of the IMP family are broadly maintained across Poaceae, despite the absence of rice from the present synteny panel.

The diverse expression patterns of the eight *SbIMP* genes under salt and osmotic stresses provide evidence for their specialized transcriptional responses to abiotic stress. Our transcriptome and qRT-PCR analyses revealed distinct temporal response strategies among family members. Specifically, *SbIMP-1*, *SbIMP-3*, *SbIMP-6*, *SbIMP-7*, and *SbIMP* - 8 function as early rapid responders to salt stress, with their expression showing strong salt-induced upregulation at 12 h after NaCl treatment. Notably, the promoter regions of these early-response genes are all enriched in multiple stress-related cis-acting elements, including ABA-responsive elements (ABRE) and MYB/MYC transcription factor binding sites (**Figure 5**). The enrichment of MYC and MYB binding sites suggests the involvement of jasmonate and ABA signaling, consistent with their established roles as key integrators in stress-responsive gene networks (Wu et al., 2026). The association between such promoter enrichment and stress-responsive expression patterns has been demonstrated in various plant gene family studies (Yamaguchi-Shinozaki and Shinozaki, 2005; Nakashima et al., 2014).

In stark contrast, *SbIMP-4*, *SbIMP-7* and *SbIMP-8* exhibited stress-specific induction, with peak upregulation at 12 h of salt stress; *SbIMP-4* was the most pronounced, suggesting a unique, non-redundant role in high-salinity signaling or ion homeostasis. The co-enrichment of DRE, ABRE and MYB binding sites in the *SbIMP-4* promoter likely constitutes the molecular basis for its salt-stress-specific expression. This unique combination of cis-elements provides a reasonable molecular explanation for strong salt-specific expression pattern of *SbIMP-4* under salt stress. Conversely, *SbIMP-2* and *SbIMP-5* displayed a delayed response pattern, showing significant upregulation only after 24 hours of PEG stress, which implies their involvement in late-stage adaptive mechanisms or cellular repair processes rather than initial sensing. This differential temporal regulation aligns perfectly with the heterogeneous distribution of stress-responsive cis-elements (such as ABRE, DRE, and MYB/MYC binding sites) identified in their promoter regions.

Such expression divergence is often accompanied by functional divergence after gene duplication. Importantly, gene duplication-driven functional divergence is widespread in plants. In Arabidopsis, IMP family members have clearly undergone functional specialization: VTC4/IMP is a bifunctional enzyme involved in both inositol and ascorbate synthesis (Torabinejad et al., 2009); *IMPL1* catalyzes the hydrolysis of inositol and galactose phosphates; and *IMPL2* is dedicated to histidine synthesis (Nourbakhsh et al., 2015; Sato et al., 2011). Similar observations have been reported in tomato, where the three IMP family members (*LeIMP-1*, *LeIMP-2*, *LeIMP-3*) differ in gene structure and expression pattern, with the LeIMP-2 promoter showing a highly specific spatial expression pattern, suggesting a unique role in inositol metabolism (Styer et al., 2004). Expression divergence after duplication has also been observed in the Poaceae RRB gene family (Chen et al., 2025). More recently, a striking example of transcriptional convergence following gene duplication was reported in barley and rice, where independent evolutionary events led to the emergence of the black hull trait through mutations in amino acid transporter genes (Li et al., 2024). Collectively, these studies indicate that functional divergence is a common evolutionary strategy following gene family expansion. For example, the sunflower Trihelix family also exhibits functional divergence under multiple abiotic stresses (Liu et al., 2026).

Given the established role of IMPase in ascorbic acid (AsA) biosynthesis, we hypothesize that the rapid upregulation of *SbIMP-1/3/6/7/8* and the specific burst of *SbIMP-4* may contribute to salt stress adaptation by boosting the AsA pool to scavenge excess reactive oxygen species (ROS), while the delayed induction of *SbIMP-2/5* may support sustained osmotic adjustment. Beyond AsA synthesis, IMPase also provides inositol for the production of membrane phospholipids. Additionally, membrane remodeling is vital for stress adaptation, as shown in barley under cold stress (Zhao et al., 2024). Similarly, SbIMP upregulation may stabilize the membrane system by modulating lipid precursor metabolism. Collectively, these findings suggest that the SbIMP family employs a multi-layered, gene-specific strategy—ranging from immediate emergency responses to delayed adaptation—to coordinate sorghum’s physiological resilience against complex environmental challenges. A similar multi-layered stress-response strategy has been observed in the C3H gene family of melon (Zheng et al., 2025).Because this study focuses on expression-level responses, stress-tolerance phenotypes (e.g., survival and growth) were not assessed under the stress conditions used; this boundary should be considered when interpreting the functional implications of the induced expression patterns. It should be emphasized that stress-induced expression does not necessarily confer stress tolerance (Xu et al., 2024), and these hypotheses require direct functional validation.

Based on systematic identification of the sorghum IMP gene family, this study reveals conserved and lineage-specific expansion features during evolution and demonstrates temporally divergent expression patterns among SbIMP members under salt and osmotic stresses. Among all family members, *SbIMP-4* stands out as the most compelling candidate for functional involvement in salt stress adaptation, given its strong salt-specific upregulation and its unique promoter architecture enriched with DRE and MYB elements. We propose that *SbIMP-4* represents a prime target for functional validation through CRISPR/Cas9-mediated knockout, transgenic overexpression, or base editing approaches (Peng et al., 2025) in sorghum, coupled with phenotypic characterization under salt stress and biochemical assays of AsA content and ROS scavenging capacity. Additionally, the early salt responders (*SbIMP-1*, *SbIMP-3*, *SbIMP-6, SbIMP-7*, and *SbIMP-8)* and late osmotic responders (*SbIMP-2* and *SbIMP-5)* warrant further investigation to dissect their respective contributions to temporal stress adaptation. Such functional studies will not only elucidate the molecular mechanisms underlying SbIMP-mediated stress tolerance but also provide actionable gene resources for precision breeding of stress-resilient sorghum varieties.

## Data Availability

The data presented in the study are deposited in the NCBI Sequence Read Archive (SRA) repository, accession number PRJNA1159475. All other data supporting the conclusions are included in the article and **Supplementary Material**.
